# Novel Splice-Altering Variants in the *CHM* and *CACNA1F* Genes Causative of X-Linked Choroideremia and Cone Dystrophy

**DOI:** 10.3390/genes16010025

**Published:** 2024-12-27

**Authors:** Anna R. Ridgeway, Ciara Shortall, Laura K. Finnegan, Róisín Long, Evan Matthews, Adrian Dockery, Ella Kopčić, Laura Whelan, Claire Kirk, Giuliana Silvestri, Jacqueline Turner, David J. Keegan, Sophia Millington-Ward, Naomi Chadderton, Emma Duignan, Paul F. Kenna, G. Jane Farrar

**Affiliations:** 1 The School of Genetics and Microbiology, Trinity College Dublin, Dublin 2, D02 VF25 Dublin, Ireland; ridgewaa@tcd.ie (A.R.R.); lafinneg@tcd.ie (L.K.F.); rolong@tcd.ie (R.L.); evmatthe@tcd.ie (E.M.); dockerya@tcd.ie (A.D.); kopcice@tcd.ie (E.K.); whelanl1@tcd.ie (L.W.); sophia@maths.tcd.ie (S.M.-W.); chaddern@tcd.ie (N.C.); paul.kenna@tcd.ie (P.F.K.); 2 Next Generation Sequencing Laboratory, Pathology Department, The Mater Misericordiae University Hospital, Dublin 7, D07 K201 Dublin, Ireland; 3 Department of Ophthalmology, The Royal Victoria Hospital, Belfast BT12 6BA, UK; claire.kirk@belfasttrust.hscni.net (C.K.); julie.silvestri@belfasttrust.hscni.net (G.S.); 4 Clinical Ophthalmic Genetics Unit, The Mater Misericordiae University Hospital, D07 K201 Dublin, Ireland; jturner@mater.ie (J.T.); dkeegan@mater.ie (D.J.K.); 5 Department of Ophthalmology, Royal Victoria Eye and Ear Hospital, Dublin 2, D02 XK51 Dublin, Ireland; emma.duignan@rveeh.ie; 6 The Research Foundation, Royal Victoria Eye and Ear Hospital, Dublin 2, D02 XK51 Dublin, Ireland

**Keywords:** choroideremia, cone dystrophy, splicing, variant of uncertain significance, functional analysis, midigene splice assays, variant interpretation

## Abstract

Background: An estimated 10–15% of all genetic diseases are attributable to variants in noncanonical splice sites, auxiliary splice sites and deep-intronic variants. Most of these unstudied variants are classified as variants of uncertain significance (VUS), which are not clinically actionable. This study investigated two novel splice-altering variants, *CHM* NM_000390.4:c.941-11T>G and *CACNA1F* NM_005183.4:c.2576+4_2576+5del implicated in choroideremia and cone dystrophy (COD), respectively, resulting in significant visual loss. Methods: Next-generation sequencing was employed to identify the candidate variants in *CHM* and *CACNA1F*, which were confirmed using Sanger sequencing. Cascade analysis was undertaken when additional family members were available. Functional analysis was conducted by cloning genomic regions of interest into gateway expression vectors, creating variant and wildtype midigenes, which were subsequently transfected into HEK293 cells. RNA was harvested and amplified by RT-PCR to investigate the splicing profile for each variant compared to the wildtype. Novel variants were reclassified according to ACMG/AMP and ClinGen SVI guidelines. Results: Midigene functional analysis confirmed that both variants disrupted splicing. The *CHM* NM_000390.4:c.941-11T>G variant caused exon 8 skipping, leading to a frameshift and the *CACNA1F* NM_005183.4:c.2576+4_2576+5del variant caused a multimodal splice defect leading to an in-frame insertion of seven amino acids and a frameshift. With this evidence, the former was upgraded to likely pathogenic and the latter to a hot VUS. Conclusions: This study adds to the mutational spectrum of splicing defects implicated in retinal degenerations by identifying and characterising two novel variants in *CHM* and *CACNA1F.* Our results highlight the importance of conducting functional analysis to investigate the consequences of intronic splice-altering variants and the significance of reclassifying VUS to confirm a genetic diagnosis.

## 1. Introduction

Choroideremia is a rare X-linked inherited retinal disease (IRD) affecting approximately 1 in 50,000 males (OMIM #303100) causing significant visual dysfunction [1]. Whilst female carriers are usually asymptomatic, they can show retinal pigment epithelial depigmentation to a variable degree due to skewed X chromosome inactivation [2]. Choroideremia is caused by variants in the *CHM* gene which encodes Rab escort protein 1 (REP-1) [3,4]. Loss of function variants in *CHM* reduce the prenylation of Rab GTPases which is believed to underpin the pathogenesis of this disease [5]. Symptoms of choroideremia typically begin in childhood and manifest as nyctalopia that gradually progresses to reduced visual acuity and visual field issues as observed in the participants of this study. To confirm a clinical diagnosis of choroideremia and enable access to future *CHM* gene therapy, identification of a likely pathogenic or pathogenic variant in the *CHM* gene is required.

Conversely, cone dystrophy (COD) is more clinically and genetically heterogeneous and is caused by variants in 40 different genes affecting approximately 1 in 40,000 people (RetNet, https://RetNet.org/ accessed on 25 April 2024) [6,7]. The participant in this study presented with progressive COD and next-generation sequencing (NGS) identified a potential splice-altering variant in *CACNA1F*. Likely pathogenic and pathogenic variants in this gene can cause progressive cone-rod dystrophy (CRD) (OMIM #300476), incomplete congenital stationary night Blindness (iCSNB) (OMIM #300071) and Åland island eye disease (AIED) (OMIM #300600) highlighting the clinical variability in IRDs [8,9,10]. The *CACNA1F* gene encodes a calcium channel α-1 subunit protein that is essential for neurotransmitter release in the phototransduction pathway [11]. Symptoms of COD include reduced visual acuity, photophobia, constriction of visual fields and colour vision defects. People with variants in *CACNA1F* may also present with nyctalopia, nystagmus, myopia, astigmatism, and a characteristic negative electroretinogram (ERG) commonly observed in *CACNA1F*-related iCSNB [12,13,14]. However, it was suggested that *CACNA1F*-related CRD is more similar to other X-linked CRDs than it is to iCSNB [8].

Splicing is an intricate process that involves converting precursor-mRNA into mature mRNA. When splicing is perturbed, aberrant transcripts may be translated into functional or partially functional proteins or more often, they may be degraded by nonsense-mediated decay (NMD). Approximately 10–15% of genetic diseases are attributable to variants that impair mRNA splicing by disrupting canonical, noncanonical, and auxiliary splice site sequence motifs [15,16]. Moreover, most non-canonical splice site variants are classified as variants of uncertain significance (VUS) due to their unknown splicing consequences and may explain a proportion of the genetically undiagnosed IRD cases. *In silico* tools are used to predict the splice-altering potential of variants with several studies reporting that SpliceAI outperforms many previously utilized tools [17,18,19]. However, to undertake a comprehensive screen for candidate splice-altering variants, a consensus approach using several tools may be optimal [19].

Variants are currently classified into five categories ranging from benign, likely benign, VUS, likely pathogenic and pathogenic according to guidelines developed by the American College of Medical Genetics and the Association for Molecular Pathology (ACMG/AMP) to establish the role of variants in an array of genetic conditions [20]. Updates and recommendations based on ACMG/AMP guidelines have been published by the ClinGen Sequence Variant Interpretation (SVI) working group to aid the variant interpretation process [21,22,23]. This involves investigation of population data, computational and predictive data, and functional data amongst other sources of evidence to assess the pathogenicity of such variants. Notably, current variant classification guidelines cap SpliceAI predictions at ACMG PP3 level for supporting evidence and is applied to variants with a score of >0.2 spliceogenicity [24]. This level of evidence may be insufficient to upgrade a VUS and, therefore, it is essential to validate *in silico* splice-altering predictions via functional analysis. Additionally, unpredicted multimodal splice defects can also arise providing rationale for functional studies to better understand the consequences of variants and their role in disease. Therefore, functional analysis using patient-derived RNA or mini/midigene assays is essential to reclassify these VUS as likely pathogenic/pathogenic making them clinically actionable.

This study investigated two novel variants in the X-linked genes *CHM* and *CACNA1F*, predicted from *in silico* analysis to be splice-altering, using midigene *in vitro* splice assays, a methodology that was used to interrogate candidate splice-altering variants causative of IRDs [25,26,27]. Our results confirmed that the *CHM* NM_000390.4:c.941-11T>G and *CACNA1F* NM_005183.4:c.2576+4_2576+5del variants resulted in aberrant splicing. Both variants were consequently upgraded from a tepid and cool VUS to likely pathogenic and hot VUS, respectively. Gathering evidence to upgrade variants is essential for patients to ensure accurate prognosis, management of the condition and eligibility for future trials and treatments.

## 2. Materials and Methods

### 2.1. Patient Recruitment

Probands and their families were recruited after clinical assessment at the Royal Victoria Eye and Ear Hospital (Dublin, Ireland) and the Royal Victoria Hospital (Belfast, Northern Ireland, UK). Following informed consent from all participants, routine clinical examination was conducted as previously described [28]. All clinical and laboratory testing was carried out in accordance with the tenets of the Declaration of Helsinki.

### 2.2. DNA Isolation and Target 5000 Next-Generation Sequencing

Patient DNA was isolated from peripheral blood using the DNA Blood Maxi Kit, Qiagen, Hilden, Germany. Sample preparation and Target Capture Next-Generation Sequencing (NGS) which encompasses the exons of 254 IRD-associated genes was performed for patients Pt-1 and Pt-7 as previously described [29]. Whole Exome Sequencing (WES) was performed for patient Pt-6. Identification of the causative variants was based on the genotype–phenotype correlation and variants were filtered as previously described to capture only those with a minor allele frequency of <1% in the Irish in-house and gnomAD (version 4) databases [30]. To confirm the candidate variants identified by NGS, the genomic regions of interest were amplified by polymerase chain reaction (PCR) using 5× FIREPol^®^ Master Mix (Cat. no., 04-11-00S25; Solis BioDyne, Tartu, Estonia) according to the manufacturer’s protocol. All primers for PCR and Sanger sequencing were designed using Primer3plus software (version 4.1.0) and purchased from Merck, Darmstadt, Germany. Annealing temperatures were obtained using UCSC *in silico* PCR software (version V39x1) and all PCR primers are available in the Supplementary Materials. Direct Sanger sequencing of the PCR products was performed by Eurofins Genomics, Ebersberg, Germany and the chromatograms were manually analysed for the presence of each variant.

### 2.3. Cascade Analysis

For family A where additional family members were available, genomic regions of interest were PCR amplified using 5× FIREPol^®^ Master Mix (Cat. no., 04-11-00S25; Solis BioDyne) according to the manufacturer’s protocols, Sanger sequenced (Eurofins Genomics, Ebersberg, Germany) and the chromatograms manually analysed for the presence or absence of each variant.

### 2.4. Variant Interpretation

Variants were classified before and after functional analysis according to ACMG/AMP criteria [20]. Variant interpretation recommendations and updates to support the use of ACMG/AMP guidelines published by the Association for Clinical Genomic Science (ACGS) and ClinGen Sequence Variant Interpretation (SVI) working group were also incorporated [21,22,23]. Sources of evidence included published population, computational, functional and segregation data. The population databases assessed were gnomAD (version 4) [30], LOVD (version 3.0) [31], ClinVar [32], Decipher (version 11.28) [33], UniProt (release 2024_06) [34] and dbSNP (build 156) [35]. The resulting candidate variants were then examined using *in silico* splice prediction tools which included SpliceSiteFinder-like [36], MaxEntScan [37], NNSPLICE (version 0.9) [38] and GENESplicer [39] accessed within Alamut Visual software version 2.1 (Interactive Biosoftware, Rouen, France; SOPHiA GENETICS, Lausanne, Switzerland) and SpliceAI (version 1.3.1) [40].

### 2.5. Midigene Generation

Variant and wildtype midigenes were generated by Long-Range PCR amplification of patient DNA using the Takara LA PCR™ Kit, Version 2.1 (Cat. no., RR013A; Takara Bio Inc., Saint-Germain-en-Laye, France) according to manufacturer’s instructions and primers with 5′ attB1 and attB2 tags to facilitate Gateway Cloning. Amplicons of 3.9 kb and 5.2 kb, respectively, were subsequently cloned into pDONOR221 vectors using Invitrogen™ Gateway Technology BP Clonase II Enzyme Mix (Cat. no., 11789021; Thermo Fisher Scientific Inc, Waltham, MA, USA) and transformed into DH10B™ competent *E. coli* (Cat. no., EC0113; Thermo Fisher Scientific Inc, Waltham, MA, USA). Plasmids were subject to diagnostic restriction digests using NheI and EcoRV and genotypes validated by Sanger sequencing. A total of 150 ng of each pDONOR221 construct was subsequently cloned into the destination vector pCI-NEO-RHO (Sangermano et al., 2016) using the Gateway LR Clonase II enzyme mix (Cat. no., 11791043; Thermo Fisher Scientific Inc., Waltham, MA, USA) [41]. Expression vectors were subject to digestion by XbaI and NheI (*CHM* midigene) and XhoI and NotI (*CACNA1F* midigene) to validate construct size and orientation and midigene inserts were Sanger sequenced.

### 2.6. Midigene Expression Analysis

HEK293 cells were maintained in prewarmed (37 °C) Dulbecco’s Modified Eagle Medium (DMEM) supplemented with 10% Fetal Bovine Serum (FBS), 1% L-Glutamine and 1% Sodium pyruvate in T75 flasks at 5% CO_2_. Cells were seeded in six well plates at a density of 5 × 10^5^ cells/well and transfected once 70% confluent with variant and wildtype midigene constructs using FuGENE HD transfection reagent (Cat. no., 32042; Active Motif, Waterloo Belgium) according to manufacturer’s instructions. At 48 h post-transfection, mRNA was isolated using the RNeasy RNA isolation kit (Cat. no., 74104; QIAGEN, Hilden, Germany).

### 2.7. RT-PCR

cDNA was generated using the iScript cDNA synthesis kit (Bio-Rad, Hercules, CA, USA) and subsequent RT-PCR performed using AmpliTaq Gold 360 DNA Polymerase (Cat. no., 4398813; Thermo Fisher Scientific Inc., Waltham, MA, USA) according to the manufacturer’s protocol. Equal quantities of RT-PCR products were analysed by 2–4% agarose gel electrophoresis, visualised via a miniBIS Pro Bio-imaging system, purified using the GeneJET PCR Purification Kit (Cat. no., K0701; Thermo Fisher Scientific Inc., Waltham, MA, USA) and Sanger sequenced. For each assay, three independent experiments were performed to ensure reproducibility. *RHO* exon 5 and 309 bp of exon 4 of the β-actin housekeeping gene were amplified by RT-PCR to assess transfection efficiency and loading of equal amounts of PCR product. Primers used are available in Supplementary Materials.

### 2.8. AlphaFold Protein Modelling and InterPro Domains

The impact of splice-altering variants on protein structure was modelled using AlphaFold 3 via the AlphaFold server [42]. Hydrogen bonds and interresidue clashes in a 5Å radius were analysed using ChimeraX (version 1.6). To predict if the frameshift transcripts were likely to undergo nonsense-mediated decay, NMDescPredictor was used [43]. The variant and wildtype proteins were analysed using InterPro (version 102.0) to detect domains [44].

## 3. Results

**Figure 1:**
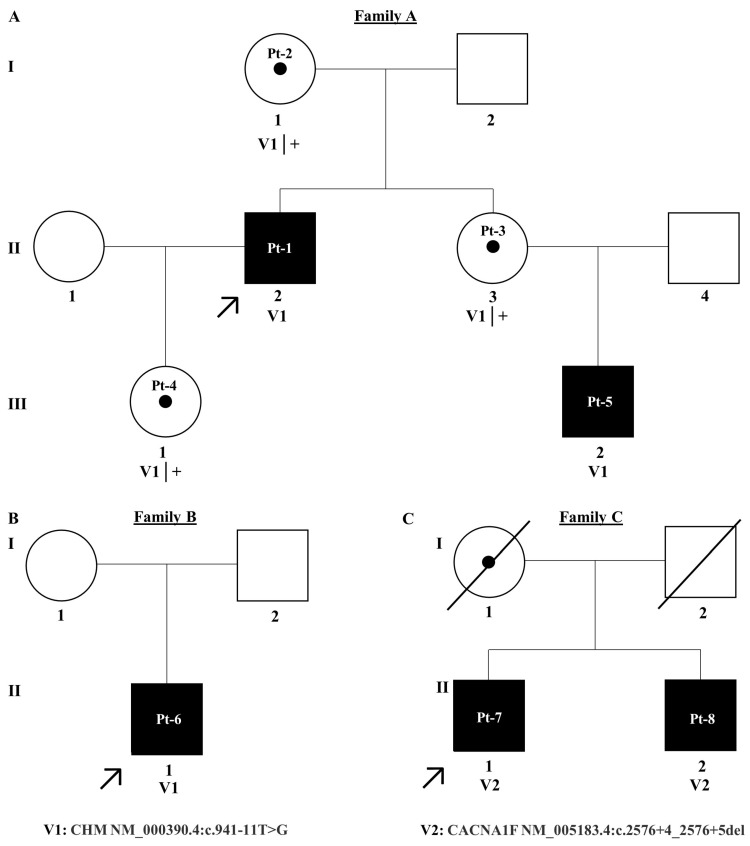
Pedigree trees for families (**A**–**C**). A = family A, B = family B and C = Family C. Affected individuals are shaded black and unaffected individuals are unshaded. Patient IDs are written within circles for females and squares for males. Probands are denoted using a black arrow. Each generation in the pedigree is denoted by I–III.

In this study, three male probands underwent NGS to identify the variant underpinning their IRD. Two VUS were detected, *CHM* NM_000390.4:c.941-11T>G, and *CACNA1F* NM_005183.4:c.2576+4_2576+5del (Figure 1). Both VUS were intronic, absent from population databases and flagged for further investigation due to their predicted effect on splicing. Therefore, *in vitro*, midigene splice assays were performed to investigate potential splicing defects and confirm their likely association with disease.

### 3.1. CHM NM_000390.4:c.941-11T>G

**Figure 2:**
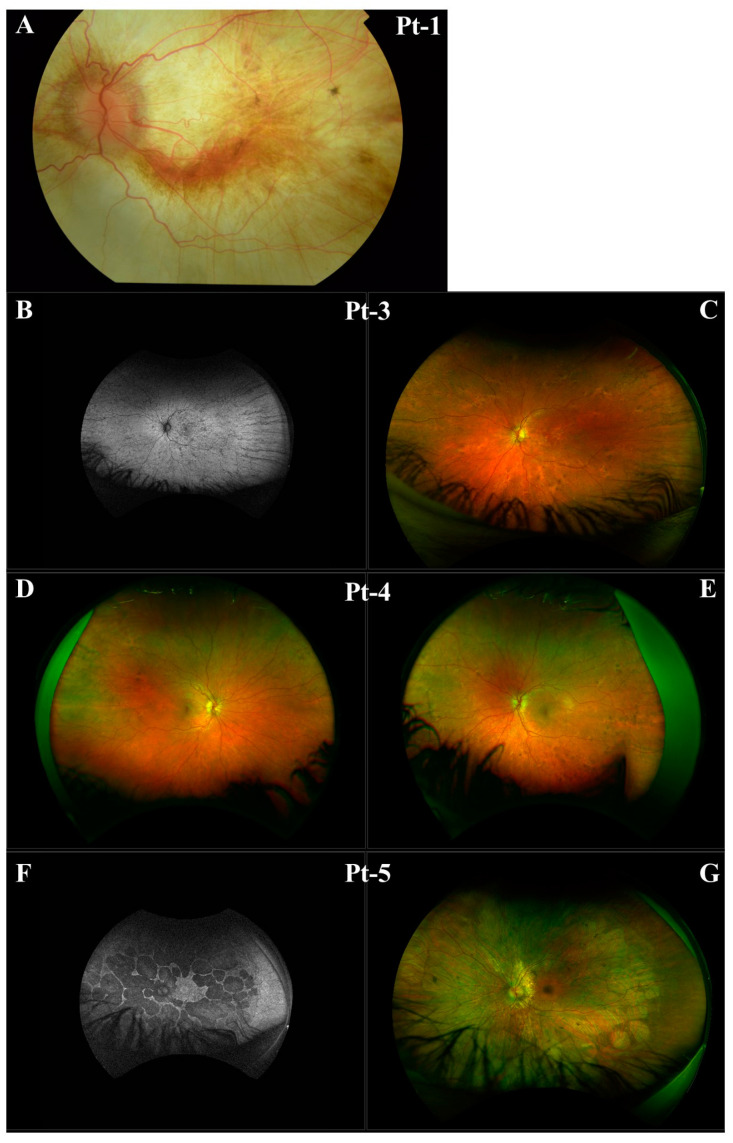
Clinical imaging for Pt-1, Pt-3, Pt-4, and Pt-5 from Family A. (**A**) Left eye fundus of Pt-1. (**B**) Left eye fundus autofluorescence of Pt-3. (**C**) Left eye fundus of Pt-3. (**D**) Right eye fundus of Pt-4. (**E**) Left eye fundus of Pt-4. (**F**) Left eye fundus autofluorescence of Pt-5. (**G**) Left eye fundus of Pt-5.

Employing NGS a non-canonical splice site variant *CHM* NM_000390.4:c.941-11T>G in intron 7 of the gene, 11 base pairs upstream of the canonical splice acceptor site, was identified in families A and B. Pt-1 developed symptoms of choroideremia at age 11 experiencing night vision issues and photophobia, which further progressed as outlined in Table 1. The proband’s sister and daughter, Pt-3 and Pt-4, were identified as carriers of the c.941-11T>G variant. Despite being asymptomatic, fundus imaging for Pt-3 and Pt-4 shows patchy marked depigmentation and RPE disturbance which is not uncommon for female carriers possibly due to skewed X-chromosome inactivation (Figure 2). The proband’s nephew, Pt-5, developed symptoms by age 6 and was also confirmed to have the c.941-11T>G variant. Pt-6, assumed to be unrelated to family A, was also hemizygous for the c.941-11T>G variant and is similarly affected by choroideremia. Of note, c.941-11T>G was the only plausible candidate variant identified in *CHM* in families A and B who have classical choroideremia. Due to the specificity of this gene as causative of choroideremia, PP4 from ACMG guidelines could be applied at supporting strength (Table 2).

*CHM* NM_000390.4:c.941-11T>G is absent from population databases and was flagged for investigation due to the high SpliceAI scores of 0.87 acceptor site loss at −11 bp, 0.45 donor site loss at −236 bp, and 0.34 acceptor site gain at −1 bp. Additionally, *in silico* tools such as SSFL, MES, NNSPLICE and GENESplicer predict that this variant is likely to perturb splicing as the acceptor site motif is destroyed.

**Figure 3:**
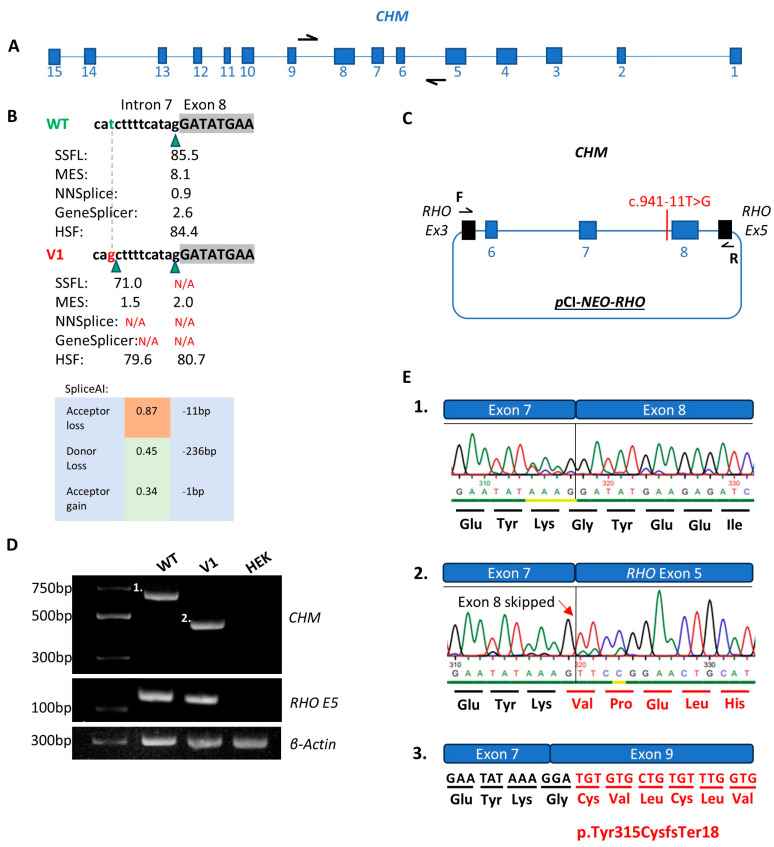
Functional analysis of the *CHM* NM_000390.4:c.941-11T>G variant (V1) compared to wildtype (WT). (**A**) Illustrates the *CHM* gene in its antisense orientation with black arrows representing the primers used to amplify the region of interest. (**B**) Scores from *in silico* splice prediction tools for WT compared to V1. Green triangles represent splice acceptor sites. The green letter t represents WT and red g represents the c.941-11T>G variant. (**C**) Schematic of the expression clone including exons 6–8 of the *CHM* gene transfected into HEK293 cells. (**D**) Agarose gel illustrating the WT (band 1 of 682bp), V1 (band 2 of 456bp), *RHO* exon 5 control for WT, V1 and HEK cell only control and β-actin control for WT, V1 and HEK cell only control. (**E.1**) Sanger sequence chromatogram from the WT purified gel product in part D band 1. (**E.2**). V1 Sanger sequence chromatogram from the V1 purified gel product in part D band 2 with the altered amino acid sequence in red text and red arrow signifying the point at which the nucleotide sequence is altered. (**E.3**). Illustration of the protein product that would result from skipping of exon 8 denoted by the altered amino acid sequence in red text.

Midigene functional analysis was conducted in HEK293 cells and transcripts of different sizes for the variant compared to wildtype identified via RT-PCR. In the variant sample (V1), a 456 bp product including exons 6–7 and *RHO* exon 5 was present. In contrast, a 682 bp wildtype product including exons 6–8 was present as expected. No residual wildtype transcript was present in the variant sample. Sanger sequencing of purified RT-PCR products confirmed that this variant results in the complete skipping of exon 8; exon skipping is commonly encountered when canonical splice acceptor sites are destroyed (Figure 3). Using HGVS nomenclature, the protein resulting from this variant is p.Tyr315CysfsTer18. The NMDescPredictor tool suggests that the transcript produced as a result of this variant is likely to undergo NMD.

**Figure 4:**
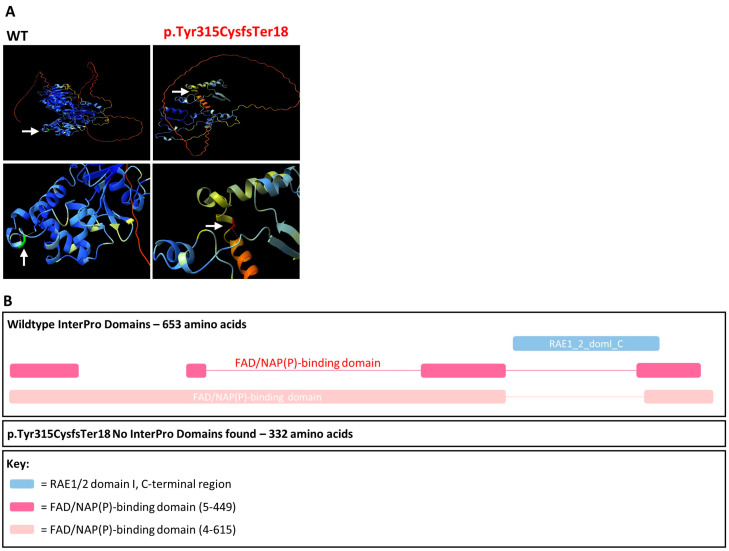
AlphaFold protein models and InterPro domains. (**A**) AlphaFold protein folding predictions for wildtype (WT) compared to the amino acid sequence produced as a result of the *CHM* c.941-11T>G, p.Tyr315CysfsTer18 variant. The Tyr315 residue is green and Cys315 residue is red. Hydrogen bonds are denoted by the orange dashed line. (**B**) InterPro domains for wildtype compared to the amino acid sequence produced as a result of the *CHM* c.941-11T>G, p.Tyr315CysfsTer18 variant.

AlphaFold protein models were generated for wildtype and truncated versions of *CHM* (Figure 4). Although the number of hydrogen bonds remains stable at residue 315, there are obvious gross structural changes to the CHM protein and as described, it is highly likely that the transcript is degraded by NMD. Additionally, InterPro identified the RAE1/2 domain I and FAD/NAP(P)-binding domain in the wildtype amino acid sequence but could not identify any domains in the truncated protein p.Tyr315CysTer18 produced as a result of the c.941-11T>G variant (Figure 4). With this evidence, the c.941-11T>G variant was reclassified as likely pathogenic scoring 6 points (Table 2).

### 3.2. CACNA1F NM_005183.4:c.2576+4_2576+5del

**Figure 5:**
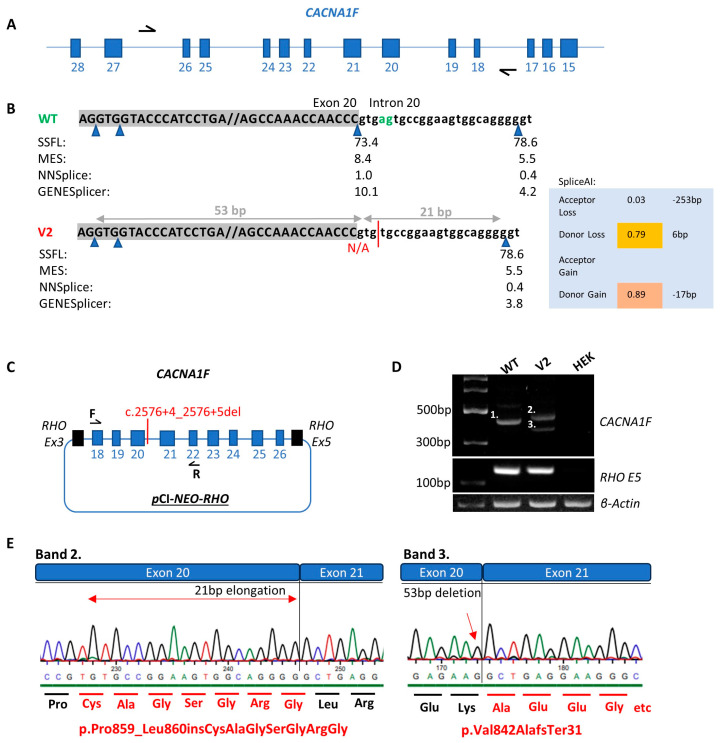
Functional analysis of the *CACNA1F* c.2576+4_2576+5del variant (V2) compared to wildtype (WT). (**A**) Illustrates the *CACNA1F* gene in its antisense orientation with black arrows representing the primers used to amplify the region of interest. (**B**) Scores from *in silico* splice prediction tools for WT compared to V2. Blue triangles represent splice donor sites. The green letters ag represent the WT nucleotide sequence and the red line represents the V2 nucleotide sequence as a result of the c.2576+4_2576+5del variant. (**C**) Schematic of the expression clone including exons 18–26 of the *CACNA1F* gene which was transfected into HEK293 cells. (**D**) Agarose gel illustrating the WT (band 1 of 418 bp), V2 (band 2 of 439 bp), V2 (band 3 of 365 bp), *RHO* exon 5 control for WT, V2 and HEK cell only control and β-actin control for WT, V2 and HEK cell only control. (**E**) Sanger sequence chromatograms from the V2 band 2 and V2 band 3 purified gel products. The altered amino acid sequence as a result of the c.2576+4_2575+5del variant is illustrated by the red text. The red arrow signifies the point at which the nucleotide sequence is altered.

Pt-7 presented in his early 20s with nystagmus and glare and was subsequently diagnosed with cone dystrophy (Table 1). A c.2543+4_2543+5delAG variant in intron 20 of the *CACNA1F* gene, four base pairs upstream of the canonical splice donor site, was identified by NGS. This variant is absent from population databases and was flagged for investigation due to the high SpliceAI scores for a 0.79 donor site loss at 6 bp and 0.89 for a donor site gain at −17 bp. Additionally, *in silico* tools such as SSFL, MES, NNSPLICE and GENESplicer predict that this variant is likely to perturb splicing as the splice donor site motif is destroyed. Midigene functional analysis confirmed that this variant, c.2543+4_2543+5delAG, results in a multimodal splice defect (Figure 5). In the wildtype, a product of 418 bp was evident as expected. In contrast, in the variant (V2), band 2 corresponded to a product of 439 bp due to a 21 nucleotide insertion, as predicted by SpliceAI and band 3 was a product of 365 bp due to a 53 nucleotide deletion in exon 20. Using HGVS nomenclature, the protein consequences could be defined as (p.P ro859_Leu860insCysAlaGlySerGlyArgGly) and (p.Val842AlafsTer31). Although the latter is predicted to undergo NMD, the resulting mature mRNA harbouring the additional seven amino acids would evade NMD. Notably, there was no residual wildtype transcript in the variant sample.

**Figure 6:**
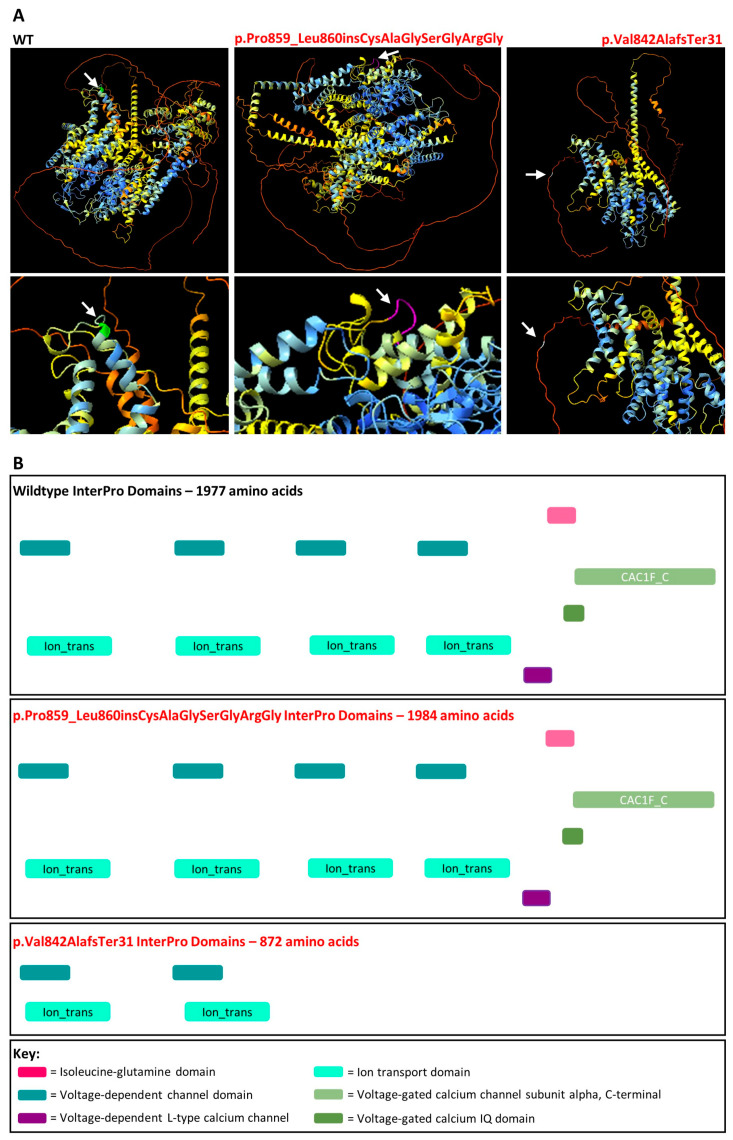
AlphaFold protein models and InterPro domains. (**A**) AlphaFold protein folding predictions for wildtype (WT) compared to the amino acid sequence produced as a result of the *CACNA1F* c.2576+4_2576+5del,p.Pro859_Leu860insCysAlaGlySerGlyArgGly or p.Val842AlafsTer31 protein change. The Pro859 residue is coloured green, Pro859_Leu860insCysAlaGlySerGlyArgGly residues are coloured purple and Ala842 residue is coloured white. The white arrows represent the region of the protein altered as a result of the protein change. (**B**) InterPro domains for wildtype compared to the amino acid sequences produced as a result of the *CACNA1F* c.2576+4_2576+5del,p.Pro859_Leu860insCysAlaGlySerGlyArgGly/p.Val842AlafsTer31 protein change.

AlphaFold protein models were generated representing the wildtype and aberrant versions of *CACNA1F* that arose due to the c.2543+4_2543+5delAG variant (Figure 6). It is clear that the incorporation of the additional seven amino acids results in a dramatic change to the overall structure of the protein and it increases the level of uncertainty in terms of predicting protein folding. However, the seven amino acids are incorporated into the cytoplasmic topological domain and are not predicted to affect the transmembrane domain or any other relevant domains when compared to the wildtype protein sequence according to UniProt and InterPro (Figure 6). Conversely, the truncated protein p.Val842AlafsTer31 results in the loss of the Isoleucine-glutamine domain, two voltage-dependent channels, two ion transporters, the L-type calcium channel, and the voltage-gated calcium channel subunit α domains. With this evidence, the c.2543+4_2543+5delAG variant was reclassified as a hot VUS scoring 5 points with the application of PVS1 at strong strength.

## 4. Discussion

In this study, two novel VUS, *CHM* c.941-11T>G and *CACNA1F* c.2576+4_2576+5del, were investigated to assess their splice-altering potential and association with the IRDs choroideremia and cone dystrophy. Functional analysis using *in vitro* midigene splice assays confirmed that both variants altered splicing and likely underpin the cause of disease for the probands in families A, B and C.

The *CHM* c.941-11T>G variant caused exon 8 skipping which was predicted by SpliceAI with a score of 0.87 acceptor site loss. This variant also creates a cryptic splice acceptor site with a SpliceAI score of 0.34. Using RT-PCR primers that bind in the expression clone backbone (Supplementary Table S1), the use of this cryptic splice acceptor site was not observed in the V1 sample. Notably, there was no residual wildtype transcript in the V1 sample (as evaluated using the exon 6–8 construct) due to the demise of the splice acceptor site illustrating the detrimental consequences of non-canonical splice site variants. The complete loss of *CHM* due to predicted NMD likely explains the severe phenotype for Pt-1. Furthermore, even if NMD was evaded, this truncated protein lacked the critical RAE1/2 domain I and FAD/NAP(P)-binding domains required for the REP-1 function [45,46]. Unfortunately, with the limited construct size of 12 kb, evaluation of the splicing of exon 7–9 was impossible because exons 8 and 9 are separated by >48 kb. For this reason, PVS1 as per latest ACMG guidelines was applied at strong strength which upgraded this variant to likely pathogenic. Therefore, obtaining patient mRNA from blood and repeating the RT-PCR would be optimal to further validate this defect to apply PVS1 at full strength.

The *CACNA1F* c.2576+4_2576+5del variant caused a multimodal splice defect as evident in Figure 5. Of note, band 3 in the V2 sample was not predicted by the *in silico* tools which underscores the importance of conducting functional analysis to fully understand the consequences of splice-altering variants. There was no wildtype transcript present in the V2 sample and the smaller band 3 resulting in the protein change p.Val842AlafsTer31 is predicted to undergo NMD (Figure 5 and Figure 6). However, the larger band 2 in the V2 sample incorporating seven additional amino acids, p.Pro848_Leu849insCysAlaGlySerGlyArgGly, is not predicted to undergo NMD. Additionally, there is no evidence that this change disrupts a critical domain in this protein as the same domains were identified by InterPro for the wildtype and p.Pro848_Leu849insCysAlaGlySerGlyArgGly amino acid sequences. Despite this, the seven additional amino acids at this position did disrupt the conformation of the folded protein compared to wildtype (Figure 6). Furthermore, a missense variant in this region, *CACNA1F* NM_005183.3:c.2579T>C,p.Leu860Pro, was classified as likely pathogenic on LOVD and observed in a patient with iCSNB [47]. Therefore, further investigation into the effect(s) of these seven additional amino acids in this region is required to explain the severe COD Pt-7 experiences. For this reason, PVS1 was applied at strong strength as the transcript resulting in p.Pro848_Leu849insCysAlaGlySerGlyArgGly may potentially be partially functional and is predicted to evade NMD.

It is important to note that the proportion of different transcripts expressed from a wildtype or mutant gene can vary between cell types due to the presence or absence of auxiliary splicing factors. Thus, it is possible that the truncated *CACNA1F* p.Val842AlafsTer31 may dominate the in-frame transcript in retinal cells which may possibly explain the progressive COD in Pt-7. However, similarly, the in-frame *CACNA1F* p.Pro848_Leu849insCysAlaGlySerGlyArgGly may have detrimental effects that have not as yet been fully elucidated. Due to the presence of universal splicing factors in this cell line and ease of transfection, HEK293 cells were used to elucidate the role of many splice-altering variants in IRDs [25,26,27]. A limitation of using HEK293 cells is that they do not exactly recapitulate the splicing process in retinal cells. For example, Albert et al., differentiated fibroblasts that were taken from patients with Stargardt Disease into photoreceptor precursor cells (PPCs) to investigate two deep intronic variants (*ABCA4* c.4539+2001G>A and c.4539+2028C>T) which cause a 345 nucleotide pseudoexon insertion resulting in a frameshift (p.Arg1514Leufs*36) [48]. Of note, this defect was not apparent in experiments using HEK293 cells. It could, therefore, be advantageous to use patient-derived PPCs or retinal organoids if available. These retinal models would also be of use for assessing VUS in genes such as *CHM* with large introns that surpass the cloning size limit.

Interestingly, Pt-7 is myopic with congenital nystagmus while their sibling, Pt-8, was found to have a characteristic electronegative ERG. These features are typical of *CACNA1F*-related iCSNB, yet the clinical diagnosis in family C was COD. Therefore, it appears the clinical features in this family are not as distinct as previously described for *CACNA1F*-related CRD [8]. This may in part be due to the multimodal splice defect caused by the *CACNA1F* c.2576+4_2576+5del variant; however, the clinical overlap between iCSNB, CRD and AIED was reported previously with no known clear genotype–phenotype correlations [13,49,50]. Identifying modifiers and better understanding the genotype–phenotype correlations in this gene is essential to offer a clear prognosis to patients and their families.

In summary, conducting functional analysis and recruiting additional family members is of paramount importance to better understand the physiological consequences of variants and potentially upgrade VUS. Based on our investigations, the *CHM* c.941-11T>G variant was reclassified as likely pathogenic and the *CACNA1F* c.2576+4_2576+5del variant as a hot VUS. Additional family members will be required to conduct segregation analysis and/or investigation into the functionality of the p.Pro848_Leu849insCysAlaGlySerGlyArgGly protein to upgrade the latter variant to likely pathogenic. Reclassifying variants in this way is essential to ensure their clinical actionability with regard to family planning and precision medicines.

## Figures and Tables

**Table 1:** Clinical information for Pt-1, Pt-4 and Pt-5-Pt-7.

Patient ID	Family ID	Status	Sex	Age of Onset	First Symptoms	BCVA	Object V Horizontal Visual Field Remaining (Goldman)	Lens	Colour Vision	Rod Response (RE)	Rod Response (LE)	Oscillatory Potential (RE)	Oscillatory Potential (LE)	Photopic (RE)	Photopic (LE)	30 Hz Flicker (RE)	30 Hz Flicker (LE)	Other
Pt-1	A	Affected	Male	11	Nyctalopia, Photophobia, RE worse than LE	RE PL, LE 6/60 age 47	RE NR, LE 20° age 47	Subcapsular Bilateral Cataracts Age 38	Pathologic Colour Discrimination	NR	NR	Reduced and Delayed Age 20	Reduced and Delayed Age 20	Significantly Reduced and Delayed Age 20	Significantly Reduced and Delayed Age 20	Significantly Reduced and Delayed Age 20	Significantly Reduced and Delayed Age 20	RE Extropia
Pt-4	A	Carrier	Female	N/A	None, Obligate Carrier	RE 6/4.8, LE 6/7.5	RE 140°, LE 135° age 19	No Cataracts	RE Diffuse Colour Error, LE Normal	Within Normal limits	Within Normal limits	N/A	N/A	Within Normal limits	Within Normal limits	N/A	N/A	RPE Changes Consistent with Obligate X-Linked CHM carrier
Pt-5	A	Affected	Male	6	Nyctalopia, Photophobia	RE 6/7.5, LE 6/7.5	RE 130°, LE 130° age 16	Cells in Vitreous Body	RE Normal, LE Diffuse Colour Error	Significantly Reduced Age 16	Significantly Reduced Age 16	NR age 16	NR age 16	Delayed and Significantly Reduced Age 16	Delayed and Significantly Reduced Age 16	Significantly Reduced Age 16	Significantly Reduced Age 16	N/A
Pt-6	B	Affected	Male	20	Nyctalopia	RE 3/60, LE 6/24 Age 50 Years	BE < 5°	No cataracts	Pathologic Colour Discrimination onset Age 40	NR	NR	N/A	N/A	Reduced	Reduced	Delayed and Reduced	Delayed and Reduced	Abnormal EOG
Pt-7	C	Affected	Male	25	Glare	RE 6/60, LE CF Age 64 Years	BE 20°	Bilateral Cataracts Age of Onset 65 Years	Normal colour Discrimination	Borderline Reduced	Borderline Reduced	N/A	N/A	Reduced	Reduced	NR	NR	Myopic, congenital nystagmus, VEP significantly delayed and borderline reduced

BCVA = best corrected visual acuity, RE = right eye, LE = left eye, PL = perception of light, NR = non-recordable, N/A = not applicable, BE = both eyes, CF = counting fingers, VEP = visual evoked potential.

**Table 2:** Classification of variants according to ACGM/AMP and ClinGen SVI guidelines. For a detailed overview of the variant interpretation, see Supplementary Table S2.

Variant	Population Data	Computational Data	Segregation Data	Other Data	Points	Classification
**Classification prior to midigene functional analysis**						
*CHM* c.941-11T>G	PM2_Supporting	PP3	N/A	PP4	3	VUS
*CACNA1F* c.2576+4_2576+5del	PM2_Supporting	PP3	N/A	N/A	2	VUS
**Classification after midigene functional analysis**						
*CHM* c.941-11T>G	PM2_Supporting	PVS1_Strong	N/A	PP4	6	Likely Pathogenic
*CACNA1F* c.2576+4_2576+5del	PM2_Supporting	PVS1_Strong	N/A	N/A	5	Hot VUS

## Data Availability

The original contributions presented in the study are included in the article/Supplementary Material, further inquiries can be directed to the corresponding author.

## Supplementary Materials

The following supporting information can be downloaded at: https://www.mdpi.com/article/10.3390/genes16010025/s1. Table S1. Primers; Table S2. Variant Interpretation before and after functional analysis.
