# Supplementary material for: Novel Splice-Altering Variants in the CHM and CACNA1F Genes Causative of X-Linked Choroideremia and Cone Dystrophy
Source: Genes (Basel). 2024 Dec 27;16(1):25. doi: 10.3390/genes16010025 (PMC11764614; doi:10.3390/genes16010025)
Supplement: Supplementary file 1 [file genes-16-00025-s001.zip › Supplementary Material_261224.pdf]

Supplementary Materials

Supplementary Table S1. Primers.

Gene	Forward (5'-3')	Reverse (5'-3')	Tm (°C)
Midigene amplification primers			
CHM	GGGGACAAGTTTGTACAAAAAAGCAGGCTT Ccaaacaaagccagagtgcct	GGGGACCACTTTGTACAAGAAAGCTGGGTGac cccacaaacacatatgcc	60
CACNA1F	GGGGACAAGTTTGTACAAAAAAGCAGGCTT Cacattctagttgaggaaggcaag	GGGGACCACTTTGTACAAGAAAGCTGGGTGgc gattctccctcctcatct	60
Sanger sequencing variant confirmation primers			
CHM c.941-11T>G	GGGAGTTCTCCTTGGCCATA	TCAACCATCAGTGTCCAAAATGT	61
RT-PCR primers			
RHO BACKBONE	acggaggtcaacaacgagtct	actggctcgtctccgtcttgg	62
CACNA1F GENE SPECIFIC	gagaagagcaatgagaaggatct	tggaggtgaaggcataatcg	58
RHO EXON 5 CONTROL	ATCTGCTGCGGCAAGAAC	AGGTGTAGGGGATGGGAGAC	58
BETA-ACTIN CONTROL	TCTTCTCCAGGGAGGAGCTG	TGCTATCCCTGTACGCCTCT	60

Supplementary Table S2. Variant Interpretation before and after functional analysis – see supplementary excel table in PDF.
